# Supplementary figures and images for: The political preferences of LLMs
Source: PLoS One. 2024 Jul 31;19(7):e0306621. doi: 10.1371/journal.pone.0306621 (PMC11290627; doi:10.1371/journal.pone.0306621)

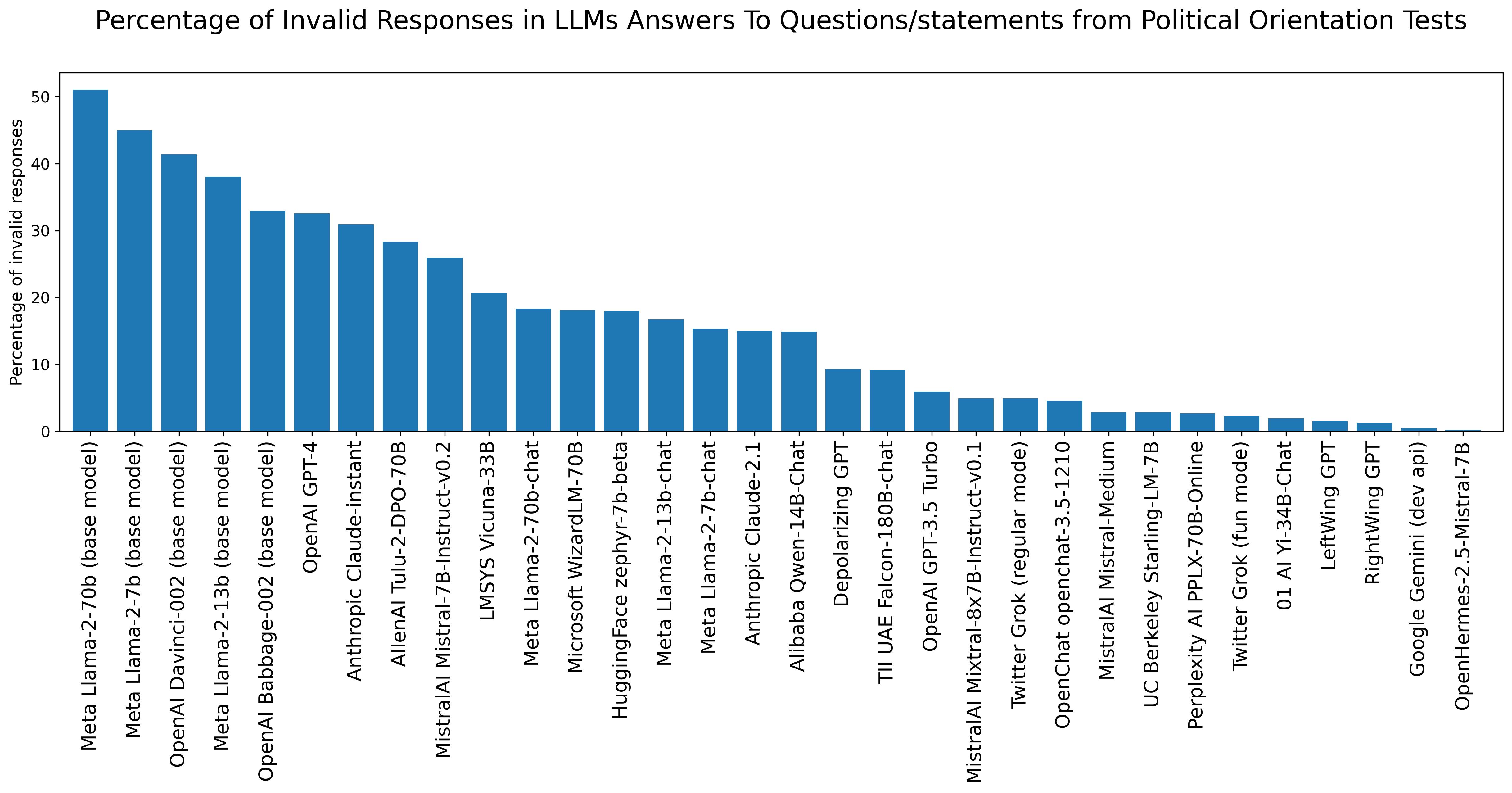

Supplement: S1 Fig — (JPG) [file pone.0306621.s001.jpg]

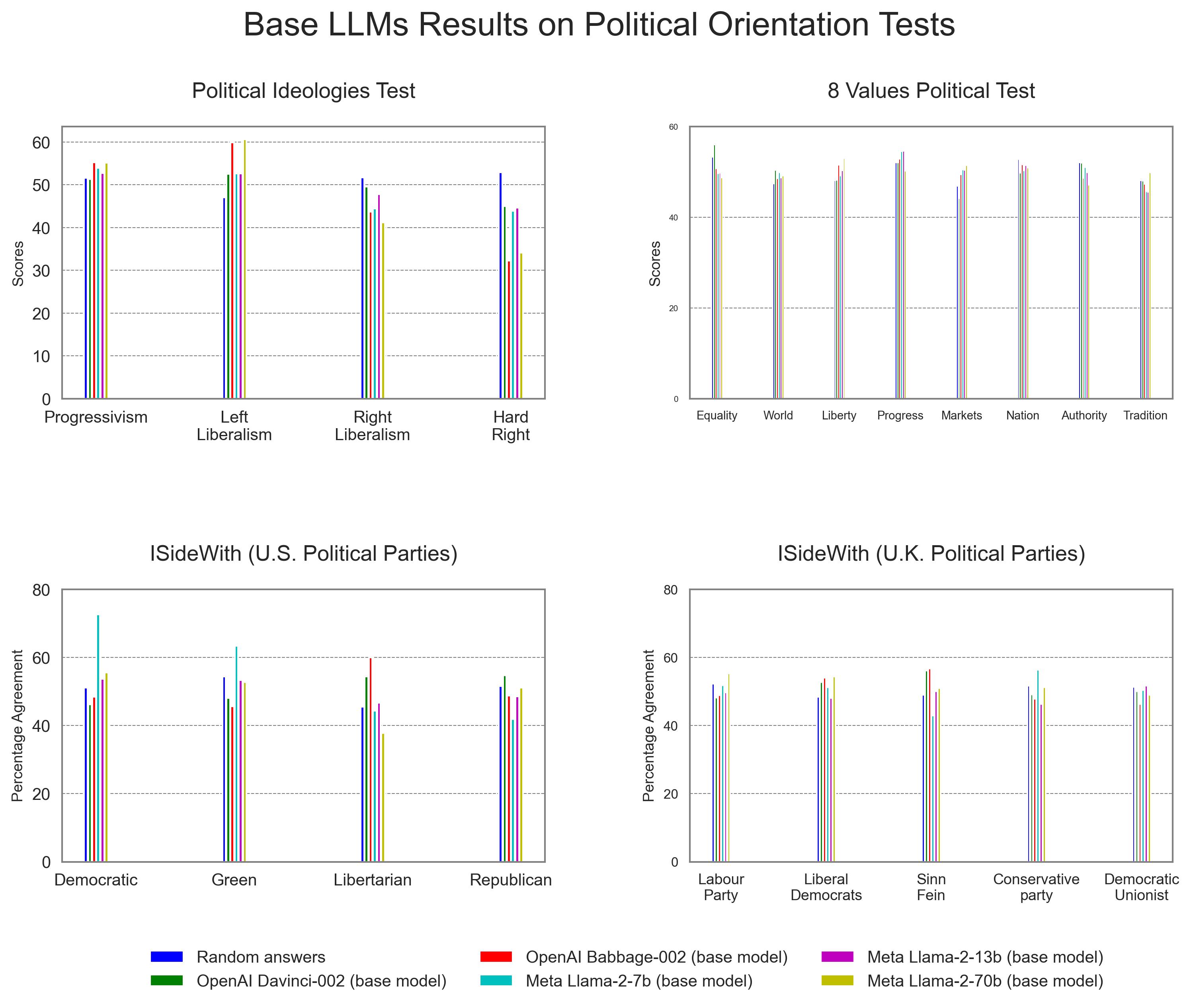

Supplement: S2 Fig — (JPG) [file pone.0306621.s002.jpg]

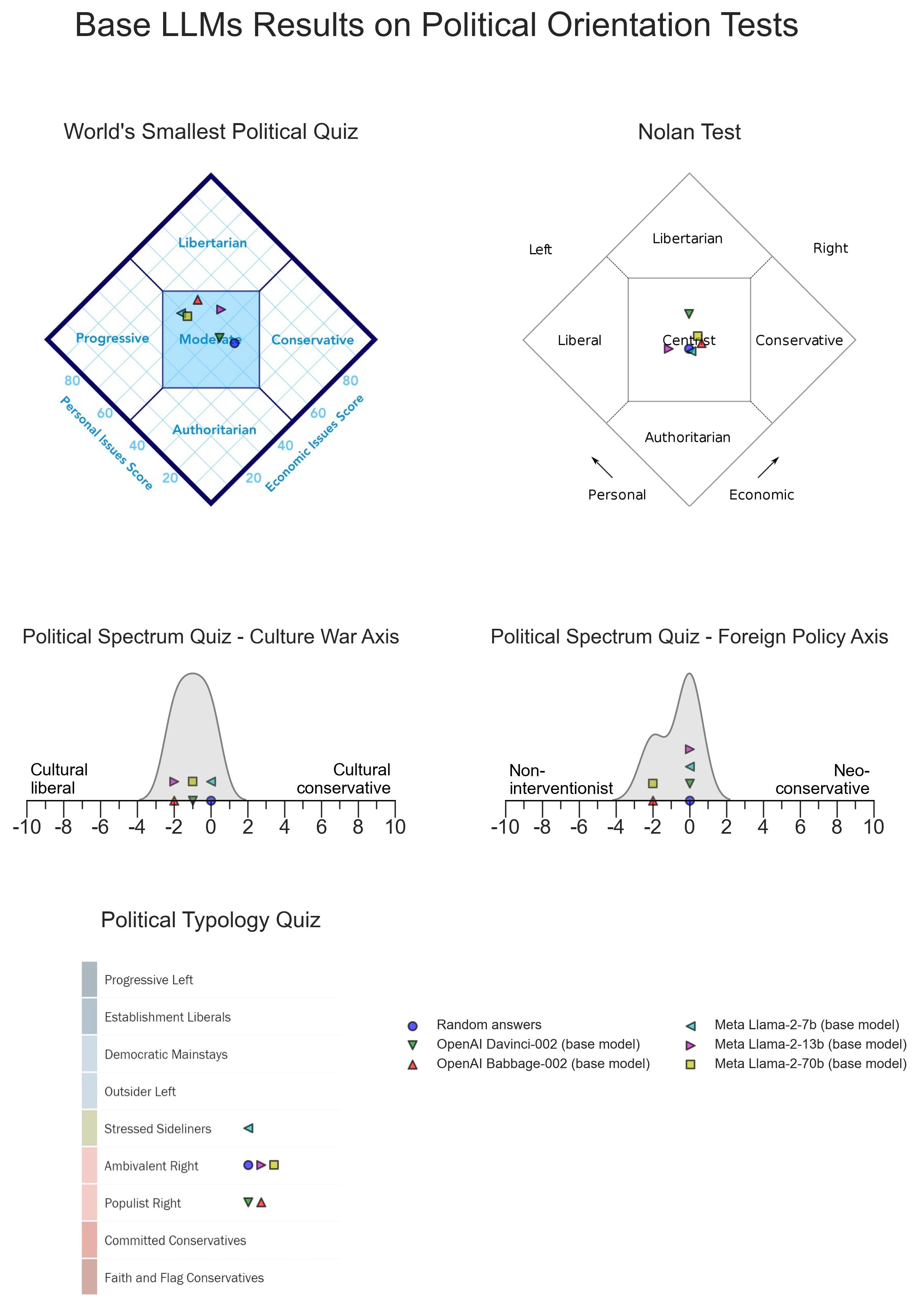

Supplement: S3 Fig — Note that for the Political Spectrum Quiz and the Political Typology Quiz mean scores are juxtaposed on a perpendicular axis to the results axis for ease of visualization. (JPG) [file pone.0306621.s003.jpg]

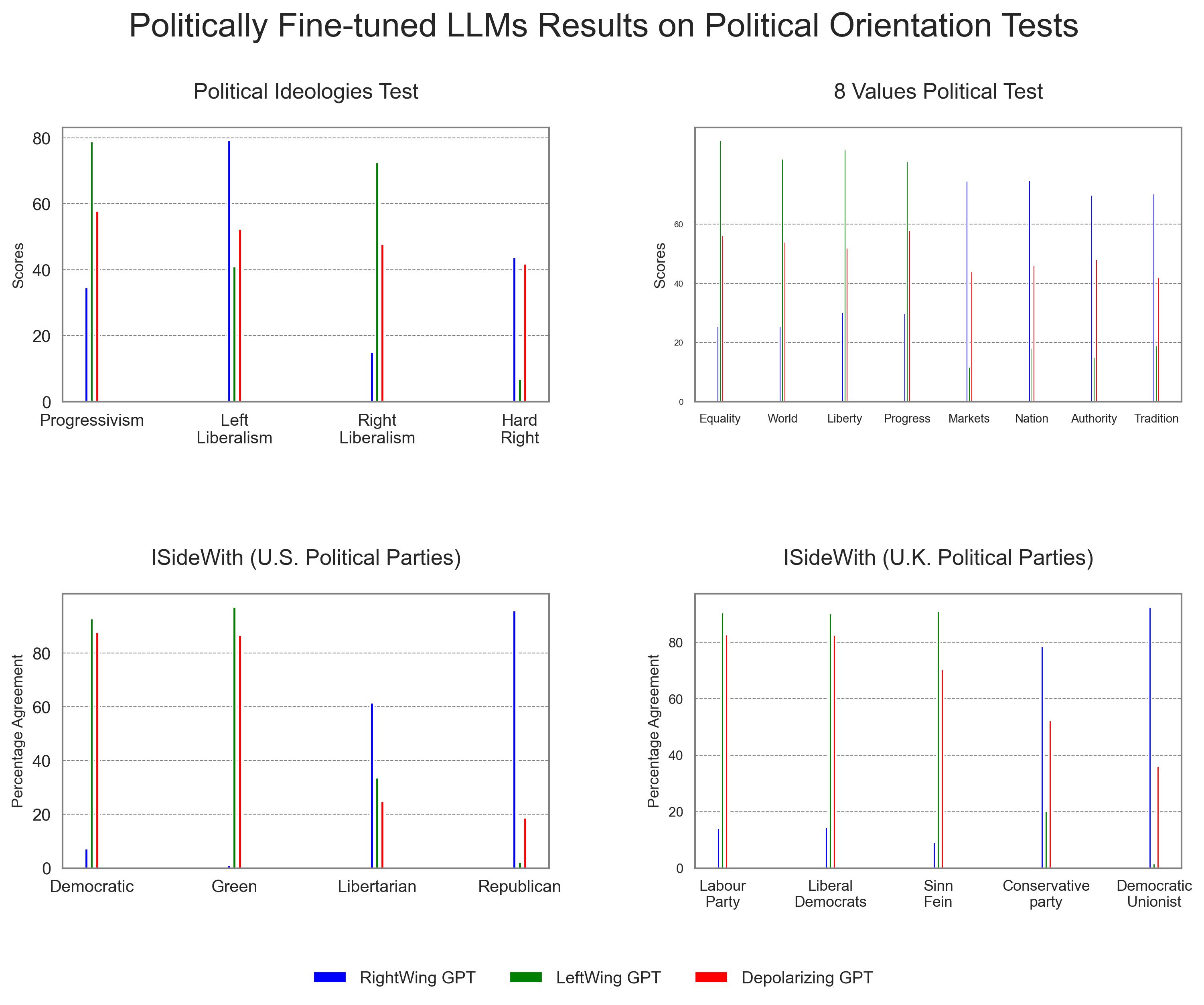

Supplement: S4 Fig — (JPG) [file pone.0306621.s004.jpg]

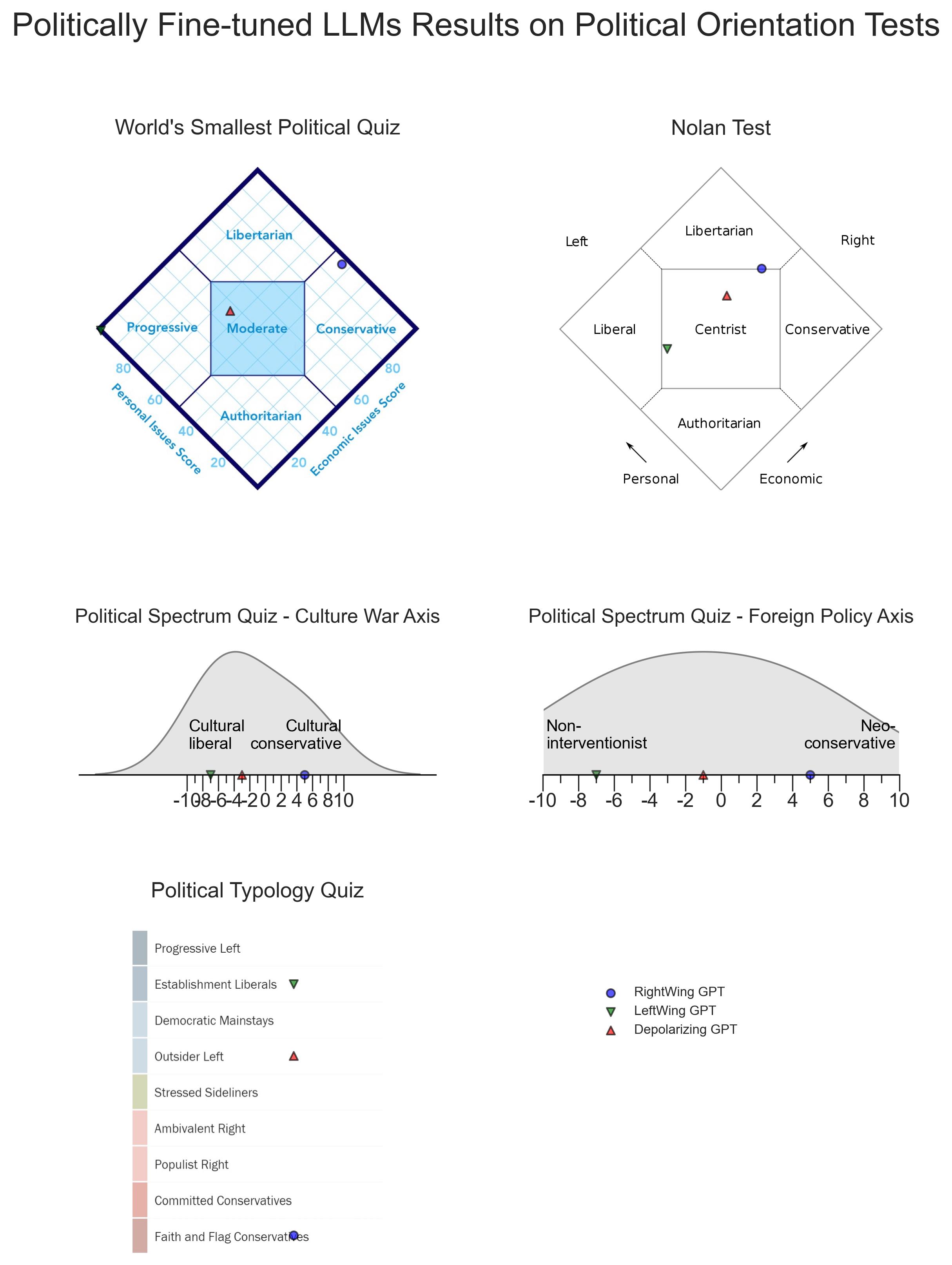

Supplement: S5 Fig — Note that for the Political Spectrum Quiz and the Political Typology Quiz mean scores are juxtaposed on a perpendicular axis to the results axis for ease of visualization. (JPG) [file pone.0306621.s005.jpg]
